# The Effect of Laser Surface Hardening on the Microstructural Characteristics and Wear Resistance of 9CrSi Steel

**DOI:** 10.3390/ma19020423

**Published:** 2026-01-21

**Authors:** Zhuldyz Sagdoldina, Daryn Baizhan, Dastan Buitkenov, Gulim Tleubergenova, Aibek Alibekov, Sanzhar Bolatov

**Affiliations:** 1 Research Center Surface Engineering and Tribology, Sarsen Amanzholov East Kazakhstan University, Ust-Kamenogorsk 070000, Kazakhstan; zh.sagdoldina@gmail.com (Z.S.); dbuitkenov@vku.edu.kz (D.B.); gulh77848@gmail.com (G.T.); aibek.alibekov.03@mail.ru (A.A.); sanzharbolatov94@gmail.com (S.B.); 2 Research School of Physical and Chemical Sciences, Shakarim University, Semey 071412, Kazakhstan

**Keywords:** laser surface hardening, 9CrSi steel, microstructure evolution, martensite, retained austenite, microhardness, elastic modulus, wear rate, dry sliding friction

## Abstract

This study presents a systematic investigation of laser surface hardening of 9CrSi tool steel with the aim of establishing the relationships between processing parameters, microstructural evolution, and resulting mechanical and tribological properties under the applied laser conditions. The influence of laser power, modulation frequency, and scanning speed on the hardened layer depth, microstructure, and surface properties was analyzed. Laser treatment produced a martensitic surface layer with varying fractions of retained austenite, while the transition zone consisted of martensite, granular pearlite, and carbide particles. X-ray diffraction identified the presence of α′-Fe, γ-Fe, and Fe_3_C phases, with peak broadening associated with increased lattice microstrain induced by rapid self-quenching. The surface microhardness increased from approximately 220 HV_0.1_ in the untreated state to 950–1000 HV_0.1_ after laser hardening, with hardened layer thicknesses ranging from about 500 to 750 µm depending on the processing regime. Instrumented indentation showed higher elastic modulus values for all hardened conditions. Tribological tests under dry sliding conditions revealed reduced coefficients of friction and more than an order-of-magnitude decrease in wear rate compared with untreated steel. The results provide a parameter–microstructure–performance map for laser-hardened 9CrSi steel, demonstrating how variations in laser processing conditions affect hardened layer characteristics and functional performance.

## 1. Introduction

High-energy surface hardening methods for ferrous alloys are aimed at forming hardened layers that enhance the wear resistance of working surfaces operating under intense mechanical loads [1]. In modern mechanical engineering, an important challenge is to improve the durability and reliability of components both during manufacturing and during the restoration of worn machine elements [2]. This necessitates the use of technologies capable of strengthening surface layers without significantly altering the bulk microstructure—an especially critical requirement for parts operating in severe service conditions [3].

Heat treatment remains a key technological process in metallurgy and mechanical engineering, ensuring the development of the required service properties of structures, tools, and machine components [4]. However, conventional quenching typically requires prolonged exposure to high temperatures, which is associated with high energy consumption, risks of distortion, and limited effectiveness when treating large or geometrically complex parts [5]. In this context, surface hardening methods that selectively modify only the functional layer without altering the core properties are attracting increasing attention [6]. Technologies such as laser hardening, induction hardening, and electrolytic-plasma treatment enable the formation of hardened layers with high hardness and wear resistance while minimizing thermal deformation of the component. Their key advantages include localized heating, high processing speed, reduced energy demand, and the capability to treat components with complex geometries. Our previous studies [7] demonstrated that electrolytic-plasma thermocyclic surface treatment can produce a hardened layer up to 700 µm thick on 30CrMnSiA steel without the formation of retained austenite. The resulting strengthened layer exhibited high structural uniformity and a pronounced martensitic transformation, confirming the effectiveness of this method for enhancing the wear resistance of structural steels. Thus, the transition from traditional bulk heat treatment to modern high-energy surface modification techniques represents an efficient approach to improving the wear resistance of ferrous alloys while preserving the mechanical integrity of the substrate.

Laser surface hardening offers extensive capabilities as a material modification technology in which a focused laser beam is used to selectively strengthen the surface of a metallic component [8]. This non-contact process provides a rapid and highly controllable heat source that is absorbed by the surface layer and induces a quench-hardening transformation without melting the substrate. The temperature of the thin surface layer must exceed the austenitization temperature for at least several tens of microseconds [9,10]. An example of laser thermal processing accompanied by similar structural transformations is laser hardening of various materials, where the formation of a melt pool is not required. In other types of laser heat treatment, melting of the surface layer does occur, such as in laser surface alloying, cladding, or amorphization. These processes result in a strengthened surface layer with excellent wear resistance and fatigue performance while maintaining the overall toughness and ductility of the core material. Current research efforts are being directed toward improving both processing parameters and resulting material performance [11].

Recent studies demonstrate that laser transformation hardening (LTH) can significantly improve the microstructure and wear resistance of tool steels. In particular, the authors of [12] investigated the influence of laser energy density on the microstructural evolution and wear behavior of pre-hardened cold-work tool steel DC53. Their results showed that increasing energy density led to pronounced grain refinement, the transformation of coarse plate-like martensite into fine needle-like martensite, and the formation of submicron carbides such as Cr_3_C_2_ and VC. At an optimal energy density of 85 J/mm^2^, the hardened layer exhibited high microstructural homogeneity, increased microhardness (up to 918 HV), and a substantial reduction in friction coefficient and wear mass loss. Moreover, the dominant wear mechanism shifted from adhesive to abrasive with increasing energy density. These findings highlight the strong interrelationship between processing parameters, microstructural transformations, and functional performance in LTH, confirming its effectiveness for improving the serviceability of cold-work tool steels.

Study [13] examined laser additive repair processing for heavily loaded railway rails, including thermal field modeling and experimental determination of optimal cladding parameters. The authors established that increasing laser power significantly affects the formation of the deposited layer, the size of the heat-affected zone, and the resulting hardness distribution. During service, the hardness of the repaired layer increased to more than 110% of the base material due to strain hardening. Contact fatigue tests showed no crack initiation or spalling after 550 kt of accumulated load, demonstrating the high potential of laser additive repair technologies for demanding railway applications. During laser hardening, the feed rate, laser power, and spot size are typically varied. Most studies employ a Gaussian intensity profile; however, transitioning to a rectangular or “chair-shaped” profile can significantly improve hardness distribution and yield sharper boundaries of the hardened zone [14]. This improvement is attributed to the more uniform temperature field generated by the modified intensity distribution, resulting in hardened tracks with narrower transition zones. Broadening the hardened zone can be achieved not only by increasing the laser spot size but also by rapidly oscillating the beam perpendicular to the feed direction [15]. Implementing such strategies requires advanced laser optics with high-speed beam control, yet offers substantial technological flexibility, including the ability to treat components with complex geometries.

Increasing the depth of the hardened layer can be achieved through multipass laser hardening [16], which provides deeper heating to temperatures above the critical point Ac_3_, where the ferrite-to-austenite transformation completes. However, increased depth is not always observed [17], and prolonged exposure of surface layers above Ac_3_ may result in grain growth and carbon homogenization [18], which can affect hardness and reduce the efficiency of the process. Laser hardening is typically performed with millisecond-range laser pulses. Using shorter pulses may lead to material ablation, whereas continuous-wave (CW) lasers require much higher power to achieve similar transformations. With the advent of high-power CW fiber lasers reaching 100 kW, larger beam spots have become available for simultaneous treatment of wide surface areas. Study [19] demonstrated the feasibility of high-productivity laser hardening using a high-power fiber laser. The authors showed that peak powers up to 120 kW with pulse durations of 10 ms increased the hardness of 42CrMo4 (AISI 4140) steel from 160 HV to 600 HV, creating a homogeneous hardened zone ~0.6 mm deep and up to 26 mm wide. The study also noted that due to high power and modulated operation, the hardening rate significantly exceeded previously reported values, and further increases in productivity can be achieved through optimization of beam shape and scanning strategy. In the study by Zhenyu Chen et al. [20], optimization of laser processing parameters resulted in a substantial increase in hardness and wear resistance of QT700-2 ductile iron. Laser hardening of structural and tool steels also presents significant scientific and practical interest due to its potential to enhance service performance in demanding applications.

Despite the extensive body of research on laser surface hardening of structural and tool steels, most published studies focus on widely used grades such as DC53, 42CrMo4, QT700-2, and AISI 4140, with an emphasis on energy density, beam shaping, or single-parameter optimization. In contrast, systematic investigations addressing the combined influence of laser power, modulation frequency, and scanning speed on the microstructure–property–performance relationships of high-carbon chromium tool steels such as 9CrSi remain limited. In particular, the role of modulation frequency in conjunction with scanning speed and power, as well as its effect on hardened layer integrity, phase composition, and tribological behavior, has not been sufficiently clarified. Moreover, comparative studies that directly link hardened layer geometry, microstructural evolution, and wear performance for 9CrSi steel under different laser regimes are scarce. This gap motivates the present work, which aims to provide a comprehensive parameter–microstructure–performance mapping for laser-hardened 9CrSi steel under controlled processing conditions.

Therefore, the aim of this work is to establish the relationships between the parameters of laser surface hardening—namely laser power, modulation frequency, and scanning speed—and the resulting microstructure, hardened layer thickness, hardness, and tribological performance of 9CrSi tool steel, as well as to determine processing regimes that provide an optimal balance between wear resistance and mechanical performance.

## 2. Materials and Methods

The substrate material used in this study was 9CrSi tool steel, which is widely employed in the production of stamping and cutting tools due to its high hardenability and wear resistance. The chemical composition of the steel, compliant with the requirements of GOST 5950-2000 [21], is characterized by the following mass fractions of the main alloying elements: C—0.85–0.95 wt.%; Si—1.2–1.6 wt.%; Mn—0.3–0.6 wt.%; Cr—0.95–1.25 wt.%. For laser surface hardening, the specimens were machined into cubic samples with dimensions of 45 × 45 × 45 mm, providing sufficient material thickness for the formation of a hardened layer while preventing thermal influence on the opposite surface [22].

Laser hardening of 9CrSi steel specimens was carried out using a Laser Quenching Machine X300-MF (Wuhan King’s Laser Co., Ltd., Wuhan, China) equipped with a semiconductor laser source with a maximum output power of 3000 W. The system incorporates an industrial robotic manipulator that ensures high-precision positioning of the laser head with an accuracy of ±0.06 mm, allowing stable control of processing parameters and the formation of a uniform hardened zone It is well known that during laser hardening, laser power, scanning speed, and pulse frequency play a decisive role in determining the depth and geometry of the hardened layer. Therefore, these parameters were varied and controlled during the experiments, while the defocus distance was kept constant. The specific laser surface hardening parameters employed in this study are summarized in Table 1. The hardening process was performed without applying any absorptive coatings on the surface (such as graphite or oxide layers), enabling the evaluation of the direct effect of laser treatment on the native steel surface [23].

Metallographic analysis of the samples was performed using an SM3200 scanning electron microscope (CIQTEK Co., Ltd., Hefei, China) operated in secondary electron (SE) and backscattered electron (BSE) modes. The specimens were etched with a 4% HNO_3_ solution in ethyl alcohol [24]. The initial microstructure of 9CrSi steel corresponds to granular pearlite with a rating of 5–6 points according to GOST 5950-2000. Phase analysis was carried out using an X’Pert PRO X-ray diffractometer (PANalytical, Almelo, The Netherlands) with CuKα radiation under the following operating conditions: tube voltage 40 kV, tube current 30 mA, exposure time 0.25 s, and step size 0.02°. The diffraction patterns were processed using HighScore software (v.3.0e) with the PDF-2 database.

Microhardness of 9CrSi steel before and after laser hardening was measured across the transverse cross-section, including the maximum depth of the hardened zone, according to GOST 9450-76 [25] using the Vickers method under a load of 0.98 N (100 g) on a METALAB 502 tester (METOLAB LLC, Moscow, Russia). Tribological tests were conducted using a ball-on-disk configuration in accordance with ASTM G99 [26] on a TRB3 tribometer (Anton Paar, Graz, Austria) under dry sliding conditions in ambient laboratory air. A ZrO_2_ ball with a diameter of 6 mm and a polished surface finish was used as the counterbody. For each testing condition, at least three independent tests were performed to ensure reproducibility, and the reported values represent averaged results. During testing, the friction coefficient was continuously recorded as a function of sliding distance, and wear measurements were performed to calculate the specific wear rate.

The geometry of the wear tracks, including profile depth and cross-sectional area, was measured using a Taylor Hobson profilometer in accordance with ISO 4287 [27]. The volumetric wear coefficient of worn samples was calculated by multiplying the length and cross-sectional area of the wear trace (1) [28]:

(1) WRs=S×lL×Fn

where:

▪ *S* is the worn track section, mm^2^;
▪ *l* is the full amplitude, mm;
▪ *L* is the total measurement distance, m;
▪ *F_n_* is the normal load, N;
▪ *WR_s_* is the wear rate of the moving sample, mm^3^ N^−1^ m^−1^.

## 3. Results and Discussion

**Figure 1:**
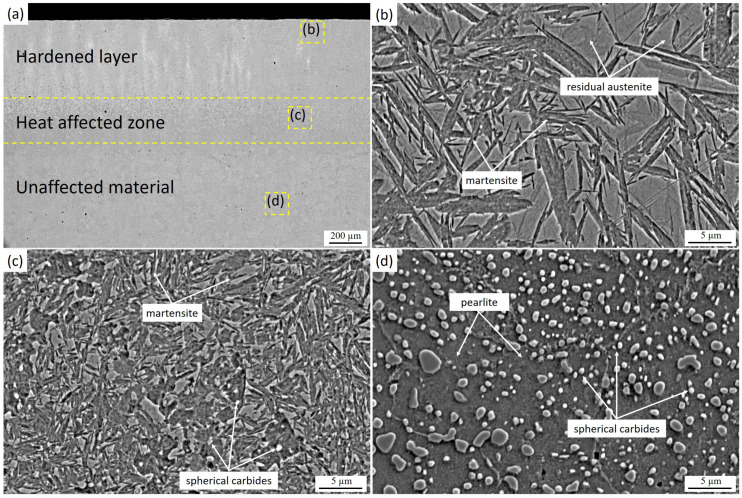
Cross-sectional microstructure of laser-hardened 9CrSi steel (LH1): (**a**) overall cross-sectional view showing the hardened layer, heat-affected zone, and unaffected base material; (**b**) microstructure of the hardened layer consisting mainly of martensite with residual austenite; (**c**) microstructure of the heat-affected zone characterized by martensite and spherical carbides; (**d**) microstructure of the unaffected base material with pearlite and spherical carbides.

Figure 1 shows the cross-sectional microstructure of 9CrSi steel after laser hardening under regime LH-1 (1500 W, 2000 Hz, 5 mm/s), demonstrating the formation of a characteristic multilayer hardened zone caused by the nonuniform heat dissipation through the material thickness. During laser treatment, the surface layer is heated to austenitization temperatures and subsequently subjected to relatively slow cooling, whereas the subsurface layers cool more rapidly due to the thermal conductivity of the bulk metal. As a result, three distinct regions are formed: a hardened surface layer, a transition zone, and the unaffected base microstructure. In the hardened layer (Figure 1b), needle-like martensite is observed along with areas of retained austenite, which is typical for high-carbon and alloy steels subjected to rapid cooling. The transition zone (Figure 1c) consists of a mixture of martensite and spheroidized carbides partially preserved after heating, reflecting the gradient in temperature and cooling rate during the laser thermal cycle. In the untreated region (Figure 1d), the original microstructure of the steel is retained, consisting of pearlite and uniformly distributed spherical carbides that did not undergo phase transformations.

After laser hardening, it was established that the microstructure of the hardened zone varies significantly depending on the processing regime. In sample LH1 (1500 W, 2000 Hz, 5 mm/s), the hardened layer consists of coarse needle-like martensite, with interlath films of retained austenite preserved between the martensite plates (Figure 1a). The thickness of the hardened layer reaches ~750 µm. As noted in [29], during heating of the hypereutectoid 9CrSi steel, carbides gradually dissolve, enriching the austenite with carbon above 0.8 wt.%, which stabilizes it and causes its partial retention after cooling. The size of the martensite plates is determined by the prior austenite grain size, which increases with the rise in heating temperature due to the system’s tendency to reduce interfacial energy by decreasing the total grain boundary area. Grain growth is further accelerated by the dissolution of carbides, which in the initial state act as pinning obstacles that limit grain coarsening.

**Figure 2:**
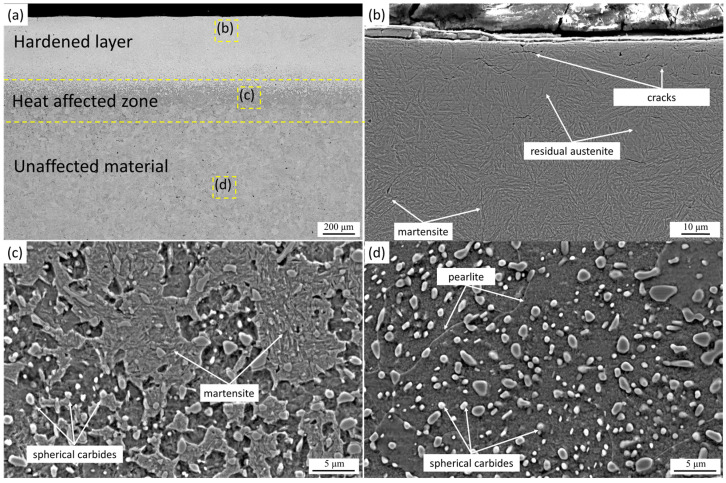
Cross-sectional microstructure of laser-hardened 9CrSi steel (LH2): (**a**) overall cross-sectional view showing the hardened layer, heat-affected zone, and unaffected base material; (**b**) microstructure of the hardened layer consisting mainly of martensite with residual austenite; (**c**) microstructure of the heat-affected zone characterized by martensite and spherical carbides; (**d**) microstructure of the unaffected base material with pearlite and spherical carbides.

Thus, the formation of coarse needle-like martensite in sample LH1 is attributed to the high austenitization temperature achieved under a relatively low scanning speed (5 mm/s), at which the thermal input per unit area is maximized despite the lower laser power compared with other regimes (Table 1). This results in more intense heating and slower cooling of the surface layers. In sample LH2 (1950 W, 2700 Hz, 10 mm/s), the hardened layer exhibits a much finer needle-like martensitic structure with a lower fraction of retained austenite (Figure 2b), while the hardened depth is approximately 570 µm. Increasing the scanning speed to 10 mm/s reduces the thermal input into the treated surface, thereby decreasing the residence time within the austenitization range, limiting grain growth, and accelerating cooling. This promotes the formation of a more refined martensitic structure. A comparison of LH1 and LH2 reveals that the key factor governing martensite morphology is the scanning speed, which directly influences the peak heating temperature and thermal gradient in the surface layer of 9CrSi steel [30,31].

An increase in the modulation frequency of the laser radiation to 2700 Hz during the treatment of sample LH2 resulted in the formation of a crack in the near-surface layers. The occurrence of cracking can be attributed to a combination of mechanical and thermal effects arising during pulsed laser processing. High-frequency modulation may generate repetitive pressure pulses that impose cyclic mechanical loading on the surface, while simultaneously enhancing thermal gradients and accelerating heating–cooling cycles. In addition to pulse-induced mechanical effects, the formation of cracks may also be influenced by transformation-induced strains associated with the martensitic transformation, as well as by the accumulation of residual stresses resulting from steep thermal gradients and constrained volume changes in the hardened layer. The characteristic orientation of the crack, perpendicular to the direction of laser beam motion (Figure 2c), suggests that non-thermal contributions, such as pulse-related stress fields, may play a role; however, thermally induced stresses and phase transformation effects cannot be excluded. The limited fraction of retained austenite in the near-surface martensitic layer further restricts stress relaxation, promoting crack initiation under combined thermal and mechanical loading. In the untreated region (Figure 2d), the steel retains its initial pearlitic microstructure with uniformly distributed spherical carbides, indicating that cracking is confined to the laser-affected zone.

**Figure 3:**
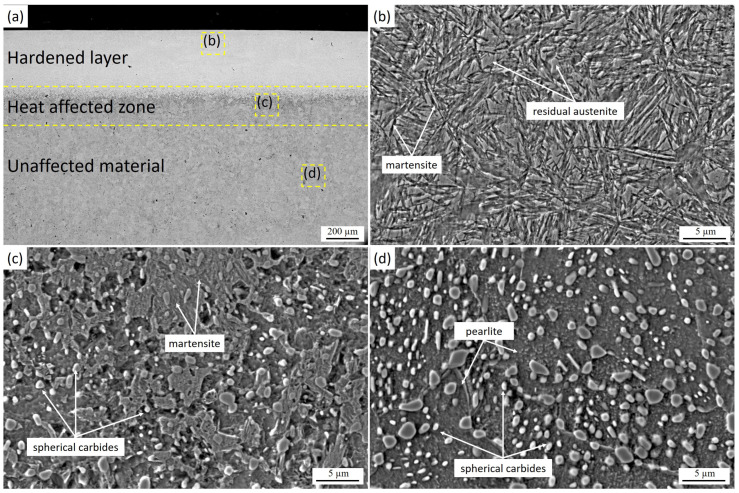
Cross-sectional microstructure of laser-hardened 9CrSi steel (LH3): (**a**) overall cross-sectional view showing the hardened layer, heat-affected zone, and unaffected base material; (**b**) microstructure of the hardened layer consisting mainly of martensite with residual austenite; (**c**) microstructure of the heat-affected zone characterized by martensite and spherical carbides; (**d**) microstructure of the unaffected base material with pearlite and spherical carbides.

In sample LH3 (2700 W, 2700 Hz, 15 mm/s), a hardened layer approximately 500 µm thick was formed, consisting of medium needle-like martensite with regions of retained austenite (Figure 3a,b). The martensite morphology is more refined compared with the coarse needle-like structure in LH1, which is associated with differences in the thermal conditions of austenitization. Despite the high laser power, the hardened depth in LH3 was smaller than in LH1 and LH2 due to the increased scanning speed of 15 mm/s, which reduces the thermal input per unit surface area and limits the depth of heating. According to the findings in [32], at high scanning speeds, the pearlitic structure can transform into high-carbon metastable martensite due to the self-quenching effect, which is consistent with the microstructure observed in LH3. Between the hardened zone and the base material, a transition layer is present, containing martensite, retained austenite, and granular pearlite (Figure 1c, Figure 2c and Figure 3c). In this region, spherical pearlite colonies and carbides partially dissolve and transform into austenite; however, due to the comparatively lower heating temperature, part of the pearlite remains, and the degree of carbide dissolution is lower than in the surface layer. This results in an increased amount of retained austenite and a reduced carbide fraction in the transition zone, consistent with the thermal gradient across the depth of the hardened layer. In the untreated region (Figure 3d), the initial microstructure of the steel is preserved and consists of pearlite with uniformly distributed spherical carbides, similar to that observed in samples LH1 and LH2.

**Figure 4:**
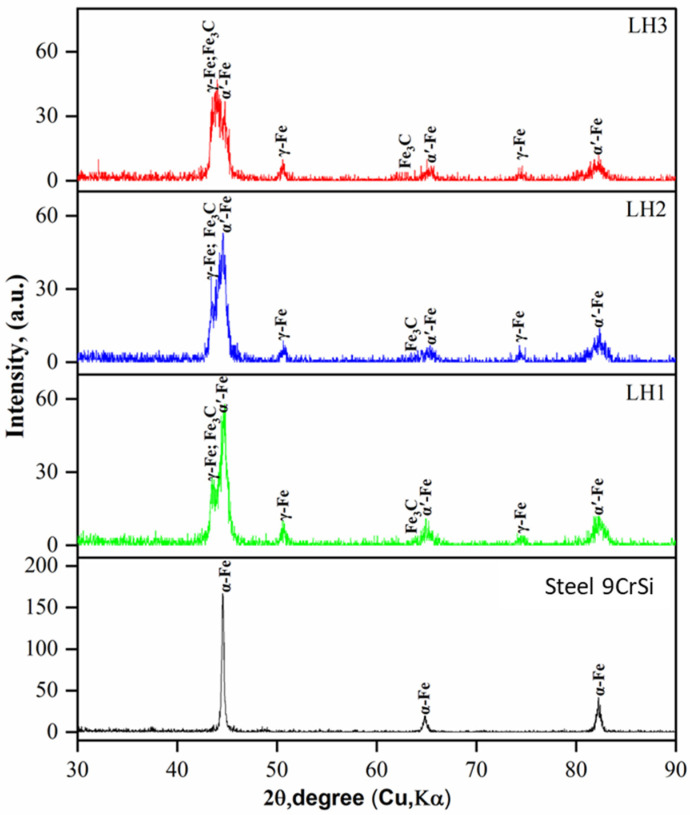
X-ray diffraction patterns of 9CrSi steel before and after laser hardening under processing regimes LH1, LH2, and LH3.

Figure 4 presents the X-ray diffraction (XRD) patterns of 9CrSi steel before and after laser hardening under different processing regimes (LH1—1500 W, 2000 Hz, 5 mm/s; LH2—1950 W, 2700 Hz, 10 mm/s; LH3—2700 W, 2000 Hz, 15 mm/s). In all examined conditions, the characteristic phases formed during hardening were identified—martensite (α′-Fe), retained austenite (γ-Fe), and Fe_3_C-type carbides—which is consistent with the metallographic observations. Notably, sample LH3 exhibits a predominance of α′-Fe peak intensities accompanied by a pronounced reduction in the γ-Fe contribution. This indicates the lowest fraction of retained austenite among the investigated regimes and confirms more extensive structural transformation resulting from the increased laser power and scanning speed.

All diffraction patterns show significant peak broadening, which is attributed to enhanced lattice microstrain and reduced coherent domain sizes produced during the rapid self-quenching inherent to laser hardening [33]. Since the breadth of diffraction maxima is sensitive to structural defects such as internal stresses, block misorientation, and dislocation density, it can be considered a reliable indicator of the degree of hardening achieved in the steel. Thus, the observed peak broadening confirms the high effectiveness of laser processing, which promotes the formation of a defect-rich martensitic structure characterized by substantial microstrain. These structural features contribute to improved mechanical and functional properties of the hardened surface layer.

**Figure 5:**
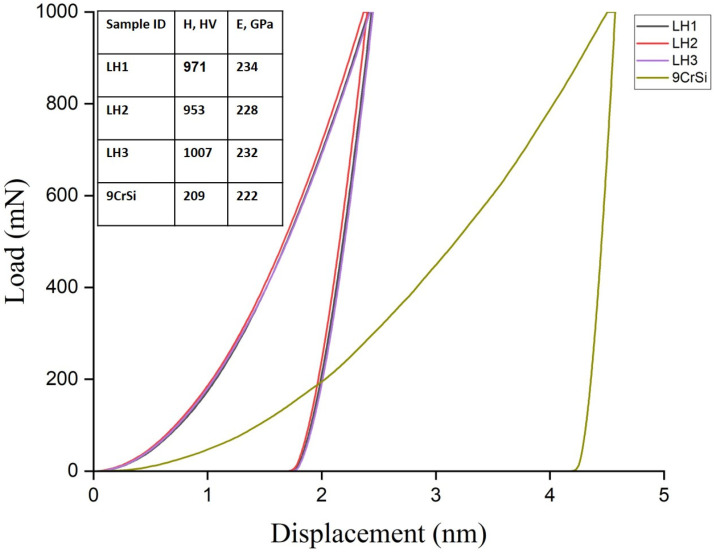
Load–displacement curves and corresponding values of surface hardness (H) and elastic modulus (E) for 9CrSi steel before and after laser hardening under different processing regimes (LH1, LH2, LH3).

Figure 5 presents the load–unload curves obtained by instrumented indentation for 9CrSi steel in the initial condition and after laser hardening under regimes LH1, LH2, and LH3, together with the corresponding hardness and elastic modulus values. All laser-hardened samples exhibit a reduced maximum indentation depth compared with the untreated steel, indicating an increased resistance to deformation associated with the formation of a martensitic surface layer. The highest hardness was obtained for sample LH3 (1007 HV_0.1_), which is consistent with the formation of a more refined martensitic structure under high laser power and scanning speed. Samples LH1 and LH2 exhibited slightly lower hardness values (971 and 953 HV_0.1_, respectively), which can be related to differences in martensite morphology and the presence of retained austenite, as confirmed by XRD analysis. The elastic modulus values of the laser-hardened samples range from 228 to 234 GPa, compared with approximately 222 GPa for the untreated steel. Although a modest increase in elastic modulus is observed after laser hardening, the differences between LH1, LH2, and LH3 are relatively small and fall within a narrow range. Considering the experimental scatter inherent to instrumented indentation measurements, these variations should be regarded as comparable rather than statistically distinct. Therefore, the elastic modulus results indicate that laser hardening leads to a general stiffening of the surface layer, while the elastic response remains largely similar across different processing regimes. In contrast to hardness, which shows a clear dependence on processing parameters and microstructural refinement, the elastic modulus appears to be less sensitive to variations in laser regime. Consequently, mechanistic interpretations based on differences in elastic modulus between individual laser-hardened conditions should be treated with caution.

**Figure 6:**
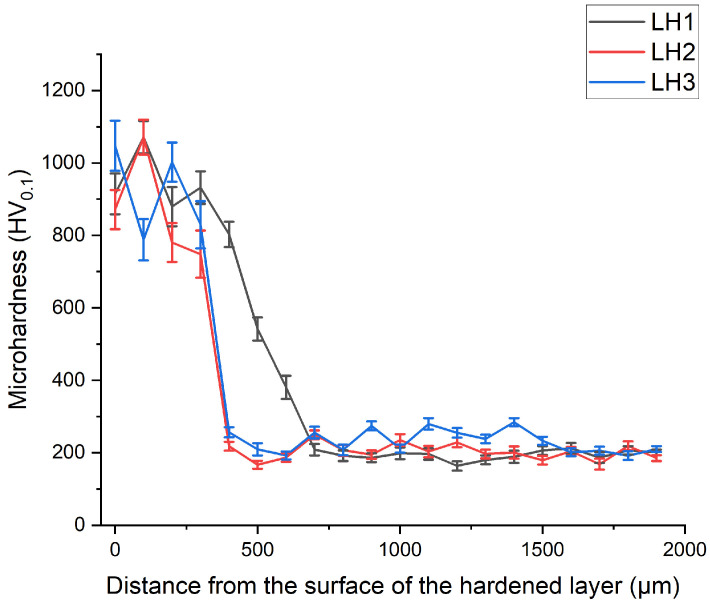
Microhardness distribution across the depth of the laser-hardened layer of 9CrSi steel under different processing regimes (LH1, LH2, LH3).

Figure 6 shows the microhardness distribution across the depth of the hardened layer of 9CrSi steel after laser processing under regimes LH1, LH2, and LH3, demonstrating a characteristic hardness gradient associated with the nonuniform thermal input and the formation of a martensitic structure in the surface region. An increase in thermal energy input at higher laser power promotes the attainment of elevated temperatures and extends the residence time of the material within the austenitization range (T > Ac_1_), thereby facilitating more intensive phase transformations [34]. The maximum microhardness values are observed in the near-surface region and reach 950–1000 HV_0.1_ depending on the processing regime. With increasing depth, the hardness gradually decreases and approaches the values characteristic of the base microstructure (~200 HV_0.1_), which is attributed to the reduced fraction of martensite and the corresponding increase in the pearlitic matrix. The smoothest hardness transition is observed for sample LH1, which is associated with the lower scanning speed and consequently higher thermal input, resulting in a wider transition zone—consistent with the microstructural features shown in Figure 1a. For hypereutectoid steel, high hardness levels are achieved under regimes that provide sufficient thermal exposure to ensure a substantial volume of martensitic transformation [35,36]. As a result of the phase transformations occurring during laser processing, the microhardness in the heat-affected zone increases from 220 HV_0.1_ to approximately 1000 HV_0.1_, corresponding to a 3–4-fold increase in hardness. Thus, the enhancement of surface hardness during laser hardening is primarily attributed to matrix strengthening associated with the transformation of the structure from pearlitic to martensitic, accompanied by a reduced fraction of retained austenite.

**Figure 7:**
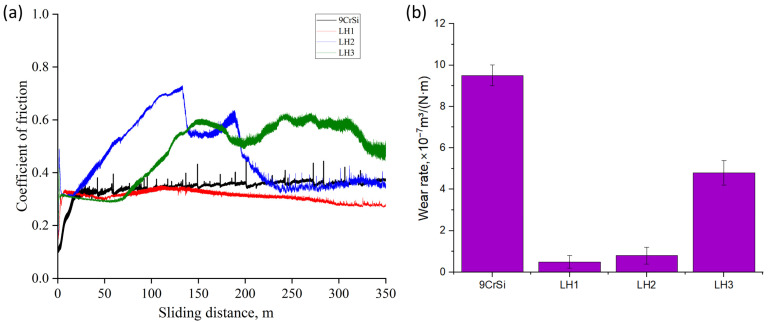
Tribological performance of 9CrSi steel before and after laser hardening under regimes LH1, LH2, and LH3: (**a**) coefficient of friction vs. sliding distance; (**b**) wear rate.

Figure 7 presents the tribological test results for 9CrSi steel in the initial condition and after laser hardening under regimes LH1, LH2, and LH3, obtained using a dry sliding configuration with a ZrO_2_ counterbody. It is evident that laser treatment significantly alters the tribological behavior of the material. The untreated 9CrSi steel exhibits a relatively high and unstable coefficient of friction, which is attributed to its pearlitic structure and lower resistance to plastic deformation under contact loading. In contrast, the laser-hardened samples show a marked reduction in the coefficient of friction and improved stability throughout the test. The lowest friction values were observed for sample LH2, which is associated with an optimal balance between martensite and retained austenite, promoting more uniform wear and reduced contact stresses. Sample LH1 demonstrates moderate friction values, whereas LH3 exhibits a higher coefficient of friction compared with LH1 and LH2, likely due to increased brittleness of the martensitic structure formed under the high thermal input. The wear-rate diagram (Figure 7b) indicates that laser hardening provides a substantial improvement in wear resistance, reducing the wear rate by an order of magnitude relative to the untreated steel. The highest wear resistance was observed for sample LH1, which can be attributed to its thicker hardened layer and smoother transition zone, enabling more uniform distribution of contact loads. Sample LH2 also shows a low wear rate, whereas LH3 exhibits slightly higher wear values, consistent with its increased friction coefficient and the possible formation of brittle surface regions. Overall, laser hardening significantly enhances the tribological performance of 9CrSi steel by reducing the coefficient of friction and increasing wear resistance through the formation of a martensitic surface layer and modification of the surface topography.

**Figure 8:**
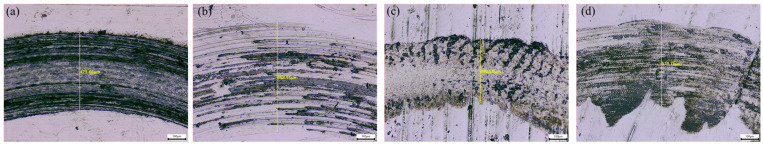
Optical micrographs of wear tracks formed after dry sliding tribological tests against a ZrO_2_ counterbody: (**a**) untreated 9CrSi steel; (**b**) laser-hardened sample LH1; (**c**) laser-hardened sample LH2; (**d**) laser-hardened sample LH3.

Figure 8 shows optical micrographs of the wear tracks formed on the surface of untreated 9CrSi steel and laser-hardened samples LH1, LH2, and LH3 after dry sliding tests against a ZrO_2_ counterbody. Clear differences in wear track morphology are observed depending on the laser hardening regime. The untreated 9CrSi steel exhibits a wide and deep wear track with pronounced plowing grooves and severe material removal, indicating intensive plastic deformation and unstable wear behavior. This morphology is consistent with the high and fluctuating coefficient of friction and the high wear rate observed for the initial pearlitic microstructure. In contrast, all laser-hardened samples show significantly narrower and shallower wear tracks, confirming the substantial improvement in wear resistance induced by laser surface hardening. For sample LH1, the wear track is relatively smooth and uniform, with shallow grooves and limited signs of severe plastic deformation. This behavior can be attributed to the thick hardened layer and smooth transition zone, which promote effective load distribution during sliding. Sample LH2 exhibits the narrowest wear track with minimal surface damage, indicating the most stable wear process. The reduced groove depth and uniform wear morphology are consistent with the lowest coefficient of friction and low wear rate observed for this regime, reflecting an optimal balance between martensite hardness and retained austenite-assisted stress relaxation. In contrast, sample LH3 shows a slightly wider wear track with localized surface damage and micro-fragmentation. This behavior is attributed to the increased brittleness of the refined martensitic structure formed under high laser power and scanning speed, which can promote microcrack initiation and material detachment during sliding. Overall, the wear track morphologies are in good agreement with the friction and wear-rate results, confirming that laser surface hardening significantly enhances the tribological performance of 9CrSi steel by reducing material removal, stabilizing the wear process, and modifying the near-surface microstructure.

## 4. Conclusions

This work systematically examined the influence of laser surface hardening parameters on the microstructure, mechanical properties, and tribological behavior of 9CrSi tool steel. The results show that laser processing leads to the formation of a hardened surface layer dominated by martensite with varying fractions of retained austenite, followed by a transition zone composed of martensite, retained austenite, and granular pearlite. The depth of the hardened layer was strongly dependent on the processing parameters, particularly the scanning speed: lower scanning speeds resulted in greater thermal input and deeper hardened layers, whereas higher scanning speeds produced thinner hardened zones.

X-ray diffraction confirmed the presence of α′-Fe, γ-Fe, and Fe_3_C phases for all hardened conditions, with peak broadening indicating increased lattice microstrain associated with rapid self-quenching. Laser hardening increased the surface microhardness from ~220 HV_0.1_ to 950–1000 HV_0.1_, while instrumented indentation revealed an increase in elastic modulus to 228–234 GPa for the hardened layers. Tribological testing demonstrated that laser hardening reduced the coefficient of friction and improved wear resistance by more than an order of magnitude compared with untreated steel.

Based on the established parameter–microstructure–performance relationships, practical technological recommendations for laser surface hardening of 9CrSi steel can be formulated. For applications requiring maximum wear resistance and a thick hardened layer, lower scanning speeds combined with moderate laser power (LH1–LH2 type regimes) are recommended, as they provide deeper hardened zones and smoother transition regions, resulting in improved load-bearing capacity and stable friction behavior. For applications prioritizing maximum surface hardness, higher laser power combined with increased scanning speed (LH3-type regime) is effective; however, such conditions should be applied with caution due to the increased brittleness and higher susceptibility to surface cracking. Overall, the results provide a practical guideline for selecting laser hardening parameters tailored to specific performance requirements of 9CrSi steel components.

## Figures and Tables

**Table 1:** Laser surface hardening processing parameters for 9CrSi steel.

Sample ID	Laser Power, W	Frequency, Hz	Scanning Speed, mm/s	Defocus Distance, m
LH1	1500	2000	5	0.4
LH2	1950	2700	10	
LH3	2700	2000	15	

## Data Availability

The original contributions presented in this study are included in the article. Further inquiries can be directed to the corresponding author.

## References

[1] Sagdoldina Z., Zhurerova L., Tyurin Y., Baizhan D., Kuykabayeba A., Abildinova S., Kozhanova R. (2022). Modification of the Surface of 40 Kh Steel by Electrolytic Plasma Hardening. Metals.

[2] Záhon L., Kuchař J., Horník J., Krčil J., Kudláček J. (2024). Laser Surface Hardening of Austempered Ductile Iron (ADI). Coatings.

[3] Bayatanova L., Rakhadilov B., Kurbanbekov S., Skakov M., Popova N. (2021). Fine Structure of Low-Carbon Steel after Electrolytic Plasma Treatment. Mater. Test..

[4] Li N., Elattar S., Xu L., Hussien M., Alqurashi Y., Saidani T. (2026). Effective Prediction of Temperature Gradient and Thermal Softening Characterizations in Heat Affected Zone in Low Frequency Pulsed Laser Assisted Turning Process of Hardened Steel Parts. Opt. Laser Technol..

[5] Rakhadilov B., Satbayeva Z., Baizhan D. Effect of electrolytic-plasma surface strengthening on the structure and properties of steel 40kHN. Proceedings of the METAL 2019—28th International Conference on Metallurgy and Materials.

[6] Rahadilov B.K., Zhurerova L.G., Sagdoldina Z.B., Kenesbekov A.B., Bayatanova L.B. (2021). Morphological Changes in the Dislocation Structure of Structural Steel 20GL after Electrolytic-Plasma Hardening of the Surface. J. Surf. Investig. X-Ray Synchrotron Neutron Tech..

[7] Baizhan D., Rakhadilov B., Zhurerova L., Tyurin Y., Sagdoldina Z., Adilkanova M., Kozhanova R. (2022). Investigation of Changes in the Structural-Phase State and the Efficiency of Hardening of 30CrMnSiA Steel by the Method of Electrolytic Plasma Thermocyclic Surface Treatment. Coatings.

[8] He C., Hu X., Qu S., Chen Z., Tang Z., Xiao H., Lin B., Lai F. (2025). Synergistic Strengthening Effect of Discrete Laser Surface Hardening and Ultrasonic Surface Rolling on the Wear and Fatigue Behaviors of Cr-Ni-Mo Steel. Surf. Coat. Technol..

[9] Sun H., Han Y., Du M., Song B., Sun Z., Lang R. (2025). Effect of Strain Hardening and Martensite Phase Transformation on Residual Stress in 30MnCrNiMo High-Strength Steel Laser-MAG Hybrid Welding. Mater. Today Commun..

[10] Satbayeva Z., Maulit A., Ispulov N., Baizhan D., Rakhadilov B., Kusainov R. (2024). Electrolytic Plasma Nitriding of Medium-Carbon Steel 45 for Performance Enhancement. Crystals.

[11] Guo S., Zhang Z.Y., Wang F.R., Jin Z.Q., Li Z.X., Yi H.L., Xie G.M. (2025). Enhanced Mechanical Properties of Laser-Oscillation Welded Al-Si Coated Press-Hardened Steel Joints with Dissimilar Thickness and Strength. Opt. Laser Technol..

[12] Zhang J., Shen S., Wu Q., Gan L., Guan C., Wang Y., Zhang Y., Zhou K., Sun S. (2025). Effect of Laser Energy Density on Microstructural Evolution and Wear Resistance of Pre-Hardened DC53 Steel by Laser Transformation Hardening. J. Mater. Res. Technol..

[13] Li J., Fan W., Zhao D., Xu J., Zhang J., Liu Z. (2026). The Influence of Laser Power on the Temperature Field and Rolling Contact Fatigue of Rail Laser Additive Repair. Eng. Fail. Anal..

[14] Fang X., Wu Y.-x., Yang X.-y., Yang Y.-g., Cheng L., Zhang Q., Liu X.-y., Mi Z.-l. (2024). Microstructure and Mechanical Properties of the Laser Welded Air-Hardening Steel Joint. Mater. Charact..

[15] Zhou Z., Lv J., Gui M., Yang W. (2024). New Insights into Annealing Induced Hardening and Deformation Mechanisms in a Selective Laser Melting Austenitic Stainless Steel 316L. Int. J. Plast..

[16] Li J., Yan H., Li S. (2023). Microstructure Characteristics at Different Depths of 40CrNiMo Steel after Laser Hardening. Mater. Charact..

[17] Liu Z., Wei Z., Zou X., Brodu E., Said D., Furtado C., Xie J., Vanmeensel K. (2024). Microstructural Evolution and Mechanical Behavior of Custom 465 Precipitation Hardening Stainless Steel Fabricated via Laser Powder Bed Fusion. Mater. Sci. Eng. A.

[18] Ding K., Wu T., Dong W., Hu T., Li S., Zhu P., Sun Y., Zhou J., Pan H., Gao Y. (2023). Clarification of the Ferrite Formed in the Laser Welded Joint of the Al–Si Coated Press-Hardened Steel. J. Mater. Res. Technol..

[19] Reich S., Heunoske D., Lueck M., Osterholz J. (2024). Laser Hardening of Steel with a 120 kW Laser at High Throughput. Procedia CIRP.

[20] Chen Z., Yu X., Ding N., Cong J., Sun J., Jia Q., Wang C. (2023). Wear Resistance Enhancement of Qt700-2 Ductile Iron Crankshaft Processed by Laser Hardening. Opt. Laser Technol..

[21] (2000). Alloy Tool Steel Bars and Strips. General Technical Conditions.

[22] Tabiyeva Y.Y., Rakhadilov B.K., Uazyrkhanova G.K., Zhurerova L.G., Maulit A., Baizhan D. (2019). Surface modification of steel mark 2 electrolytic plasma exposure. Eurasian J. Phys. Funct. Mater..

[23] Dumitrescu P., Koshy P., Stenekes J., Elbestawi M.A. (2006). High-Power Diode Laser Assisted Hard Turning of AISI D2 Tool Steel. Int. J. Mach. Tools Manuf..

[24] Skvarenina S., Shin Y.C. (2006). Laser-Assisted Machining of Compacted Graphite Iron. Int. J. Mach. Tools Manuf..

[25] (1976). Measurement of Microhardness by Indentation of Diamond Tips.

[26] (2017). Standard Test Method for Wear Testing with a Pin-on-Disk Apparatus.

[27] (1997). Geometrical Product Specifications (GPS)—Surface Texture: Profile Method—Terms, Definitions and Surface Texture Parameters.

[28] Rakhadilov B., Kengesbekov A., Zhurerova L., Kozhanova R., Sagdoldina Z. (2021). Impact of Electronic Radiation on the Morphology of the Fine Structure of the Surface Layer of R6M5 Steel. Machines.

[29] Łach Ł. (2024). Recent Advances in Laser Surface Hardening: Techniques, Modeling Approaches, and Industrial Applications. Crystals.

[30] Maharjan N., Wu N., Zhou W. (2021). Hardening Efficiency and Microstructural Changes during Laser Surface Hardening of 50CrMo4 Steel. Metals.

[31] Karamimoghadam M., Rezayat M., Moradi M., Mateo A., Casalino G. (2024). Laser Surface Transformation Hardening for Automotive Metals: Recent Progress. Metals.

[32] Barka N., Sattarpanah Karganroudi S., Fakir R., Thibeault P., Feujofack Kemda V.B. (2020). Effects of Laser Hardening Process Parameters on Hardness Profile of 4340 Steel Spline—An Experimental Approach. Coatings.

[33] Babič M., Marinkovic D., Bonfanti M., Calì M. (2022). Complexity Modeling of Steel-Laser-Hardened Surface Microstructures. Appl. Sci..

[34] Borki A.K., El Ouafi A., Chebak A. (2019). Experimental Investigation of Laser Surface Transformation Hardening of 4340 Steel Spur Gears. J. Manuf. Mater. Process..

[35] Paczkowska M. (2024). Modification of the Surface Layer of Grey Cast Iron by Laser Heat Treatment. Lubricants.

[36] Zhang Q., Ling J., Chen Z., Wu G., Yu Z., Wang Y., Zhou J., Yao J. (2025). Optimization of Laser-Induced Hybrid Hardening Process Based on Response Surface Methodology and WOA-BP Neural Network. Appl. Sci..

